# Barriers and facilitators to training delivery and subsequent implementation of a localised child and adolescent mental health initiative: a qualitative content analysis

**DOI:** 10.1186/s12909-023-04238-9

**Published:** 2023-04-19

**Authors:** Emily Banwell, Pamela Qualter, Neil Humphrey

**Affiliations:** Institute of Education, University of Manchester, Ellen Wilkinson Building, Manchester, M13 9PL UK

**Keywords:** Child and adolescent mental health, Evaluation, Implementation science, Professional development, Qualitative content analysis, Training, Barriers and facilitators

## Abstract

**Background:**

Ensuring that children and young people (CYP) can obtain mental health support from a broad variety of sources is of upmost importance. This is especially true given the increasing prevalence of mental health difficulties in this population, and the associated challenges with receiving support from specialised healthcare services. Equipping professionals, from a wide range of sectors, with the skills needed to provide this support is a vital starting point. This study explored the experiences of professionals who had participated in CYP mental health training modules that related directly to the local implementation of the THRIVE Framework for System Change in Greater Manchester, UK (GM i-THRIVE) to establish the perceived barriers and facilitators behind the implementation of this training programme.

**Methods:**

Directed qualitative content analysis of semi-structured interview data from nine CYP-facing professionals was conducted. Both the interview schedule and initial deductive coding strategy were developed using the findings of a systematic literature review by the authors, that was conducted to explore wider CYP mental health training experiences. This methodology was used to establish the presence or absence of these findings within GM i-THRIVE, before generating tailored recommendations for their training programme.

**Results:**

When the interview data were coded and analysed, a strong level of thematic similarity with the authors’ review was found. However, we deduced that the emergence of additional themes might reflect the contextual uniqueness of GM i-THRIVE, that is likely to be further compounded by the COVID-19 pandemic. Six recommendations were made for further improvement. These included the facilitation of unstructured peer interaction during training, and ensuring that jargon and key words are fully clarified.

**Conclusions:**

Methodological limitations, guidance for usage, and potential applications of the study’s findings are explored. Whilst the findings were largely akin to those of the review, subtle yet important differences were found. These are likely to reflect the nuances of the training programme discussed, however, we tentatively suggest that our findings are transferable to similar training interventions. This study provides a valuable example of how qualitative evidence syntheses can be used to aid study design and analysis: an underused approach.

**Supplementary Information:**

The online version contains supplementary material available at 10.1186/s12909-023-04238-9.

## Introduction

### Background

One in six 6–16 year-olds (17.4%) in England had a probable diagnosable psychiatric disorder in 2021: a concerning increase from the one in nine (11.6%) reported in 2017 [1]. Given that the peak age of onset for psychiatric disorders is 14.5 years old [2], the need for the earliest possible intervention is clear. Despite this, many CYP are unable to access appropriate mental health support. Funding cuts to Child and Adolescent Mental Health Services (CAMHS) [3], lengthy waiting times [4], and high referral rejection rates [5] are all plausible explanations for why specialist support is inaccessible to many. Referrals are often considered inappropriate, and are therefore rejected, when CYP do not meet a diagnostic threshold in terms of symptoms or severity [6].

Although efforts *are* being made to improve CYP access to specialist NHS services [7, 8], there remains an obvious need for alternative provision of support. CYP already rely on the various non-mental health trained professionals that they encounter in their day-to-day lives for support and advice, with teachers being particularly valued sources [9]. At times of mental health crisis, Accident and Emergency (A&E) departments are frequently a port of call, despite being poorly equipped for psychiatric admissions [10]. Teachers, similarly, feel under-trained in this area: he time and resources needed to provide an ideal level of mental health support are simply not available to them [9]. Even GPs lack the expertise needed to both support and refer appropriately when it comes to mental health. These shortcomings have been acknowledged by GPs themselves [11, 12], as well as CYP reporting that they do not feel comfortable approaching GPs for these reasons [13–15].

### THRIVE – a nationwide initiative

As an initiative aiming to remedy some of the shortcomings of current CYP mental health services, THRIVE [16] has so far been introduced in over 70 areas in England. The THRIVE framework epitomises a holistic view of mental health care, meaning that anyone who encounters CYP in a professional capacity, for example through school, social care, the criminal justice system, or even the arts sector, will be equipped with the level of training and knowledge needed to act as informed advisers in times of mental health need. Within CAMHS specifically, THRIVE aims to improve cross and within-sector communication, meaning that accountability becomes shared. This will hopefully build a more effective service for those requiring specialist care. The fact that THRIVE represents a common-language framework means that a consistent service should be provided by all THRIVE-trained professionals. For CYP, this means that there will never be a ‘wrong door’ in which to turn [17]. In all, those unable to access specialist CAMHS, for whatever reason, should have a diversified range of options through which to receive assistance. Research into the impact that THRIVE has had on the provision of support so far, from the perspectives of key stakeholders, [18] has highlighted the importance of factors such as needs-based support, making decisions alongside service users, and interagency working, for improving the accessibility and quality of care. The study indicates that system-wide support for these principles would further enhance their benefits [18]. Another study, that examined a THRIVE implementation site in London [19] suggested that THRIVE has resulted in a more efficient service, where resources are used more effectively and flexibly. The study concluded that the new structure meant that more staff and service users were able to benefit from pockets of expertise within the system. This final point in particular suggests that in order to make the goals of the THRIVE framework a reality in Greater Manchester, a wide-spread, comprehensive training agenda is necessitated.

### Greater manchester – an implementation site

The implementation of the THRIVE framework in Greater Manchester (known locally as GM i-THRIVE) commenced in 2018, and represents part of a wider devolution deal drawn between the Greater Manchester Health and Social Care Partnership (GMHSCP) and the UK government. This devolution allowed the region to make its own decisions about how local NHS services are funded [20], based on the needs of the 2.8 million city-region residents. The core GM i-THRIVE team work with leaders in each of Greater Manchester’s ten locality boroughs (Bolton, Bury, Manchester, Oldham, Rochdale, Salford, Stockport, Tameside, Trafford, and Wigan) to align the CYP mental health services within each, including CAMHS, voluntary sector, and wider CYP-facing, to THRIVE’s principles [21]. To do this, four key training modules have been developed, for what is known as the GM i-THRIVE Training Academy. These were designed to facilitate implementation, allowing services to equip their workforce to deliver THRIVE’s objectives. The four training modules are as follows:

1. **“Shared decision making”**: trainees learn the importance of having conversations about treatment or care *alongside* CYP and their families, to make joint decisions.
2. **“Getting advice and signposting**”: trainees learn how to signpost effectively and efficiently to other services or help sources.
3. **“Getting risk support**”: trainees learn to recognise the needs of CYP and families that are at risk of harmful experiences, and how these experiences can relate to mental health outcomes. This is especially important in cases where CAMHS services have thus far been unable to elicit a positive change for a particular individual. It helps professionals to safeguard effectively, and to use methods that support multi-agency working.
4. **“Building confidence in letting go and managing difficult endings”**: Ending therapeutic support is difficult for CYP and those helping them. This module discusses what makes these endings challenging, and helps professionals to instigate an open dialogue with CYP about what successful and realistic therapeutic outcomes look like.

The training modules are broadly accessible to a wide variety of professionals, so that a comprehensive system of support for CYP can be built [21]. These include CAMHS staff, local authority, and educational professionals. From 2019 onwards, the training was held face-to-face, repeated in geographically accessible locations for those working in any of the ten Greater Manchester localities. However, after the outbreak of COVID-19, training was moved to an online format, comprising both synchronous and asynchronous content.

The authors of the present study are involved in evaluating GM i-THRIVE, part of which is an investigation of the barriers and facilitators underpinning successful CYP mental health training delivery and implementation. To identify whether such factors had been explored in other qualitative studies with professionals who had completed similar training, we recently conducted a systematic literature review (SLR) and qualitative meta-synthesis [22]. In the review, we searched the literature for qualitative studies, whereby participants discussed their experiences with training designed to improve their knowledge of CYP mental health. These studies included both participants who had previous mental health training, plus allied professionals who had not. The resulting findings were then synthesised using qualitative meta-aggregation, and we made nineteen practical recommendations for those designing, delivering, or implementing such training (see Table 1). These ranged from highlighting the importance of training support, to ensuring that the training is needed and appreciated within the implementing organisation. The paper drew what were essentially ‘common-sense’ principles, from a strong evidence base. They were then tied, using a pragmatic methodology, into a coherent and accessible framework.

**Table 1:** An exhaustive list of the themes and codes that represented the data in the present study, and the number of extracts pertaining to each. Phrases in brackets refer to the names that were given to themes in the authors’ SLR [22]

Themes and codes	Extracts	Number of inductive codes per theme
**Peer support (with a little help from my friends)**	**37**	4
*Trainees from different professional backgrounds sharing ideas and experiences*	*13*	
Provided opportunities to interact*	*8*	
Make connections with similar people*	*6*	
Encourage conversation*	*5*	
*Reduce feelings of isolation*	*4*	
Learning about problems in the wider sector*	*1*	
**Does it reflect reality? (keep it real)**	**32**	**3**
*Cover and discuss the trainees’ own workplace challenges*	*7*	
*Use real-world examples*	*6*	
*Link closely to real-world delivery and implementation*	*4*	
Dealing with complex cases*	*4*	
Patient point of view explored*	*4*	
*Applicable to the implementing environment*	*3*	
*Feasible implementation*	*2*	
Theory to practice*	*2*	
**Suitability (know your audience)**	**31**	**5**
*Consider the diverse backgrounds of trainees*	*8*	
*Appropriate content*	*8*	
*Accessibility*	*4*	
Training builds upon previous knowledge*	*4*	
Design process*	*3*	
The sequencing of the training modules*	*2*	
Gaps in knowledge are easy to identify*	*1*	
Inclusivity*	*1*	
**In-training support (in the moment)**	**28**	**1**
*Feedback*	*12*	
*Dialogue rather than passive listening*	*9*	
*Logistical and practical supports*	*5*	
Within training resources*	*2*	
**Everyone on board (are we on the same page?)**	**23**	**2**
*The entire organisation should be ‘on board’*	*15*	
System-wide implementation*	*6*	
Training informs about current and relevant issues*	*2*	
*A supportive environment*	*0*	
**Timing (pace yourself)**	**20**	**1**
*Last an appropriate duration*	*10*	
*Appropriate amount of information*	*8*	
Prep work was needed*	*2*	
Expectations versus reality*	**19**	**2**
Reasons for attending*	*13*	
Did it match your expectations?*	*6*	
**Changing mind-sets (get in their heads)**	**17**	**1**
*An appropriate level of background knowledge may be needed for maximum gains*	*9*	
Is this any of my business?*	*4*	
*It can be difficult to change trainees’ habits*	*2*	
*Encourage a change of mind-set*	*2*	
**Leadership qualities (lead the way)**	**14**	**2**
*Experts in the topic*	*7*	
*Relatable and understanding*	*5*	
Multiple trainers present*	*4*	
Support from ‘above’*	*3*	
**Flexible application (expect the unexpected)**	**14**	**2**
*Appropriate for the reality of the implementing environment*	*6*	
Dealing with complex cases*	*4*	
*Acceptable to the population that it aims to help*	*3*	
Are we allowed to be flexible?*	*1*	
Issues relating to the COVID-19 pandemic*	**13**	**2**
Issues specific to the online training environment*	*12*	
Struggles with gaining a place because of COVID-19*	*1*	
**Smooth and seamless (blending in)**	**13**	**1**
*Link with current practice*	*5*	
Training builds upon previous knowledge*	*4*	
*Bring other practices together*	*3*	
*Smooth and seamless*	*1*	
**Confidence and capability (power to the people)**	**12**	**1**
*Build trainee confidence*	*6*	
*Improve trainee competence*	*5*	
Signposting knowledge increased*	*1*	
**Broad reach (cast a wide net)**	**11**	**0**
*Empower trainees to disseminate their learning*	*7*	
*Advertised or offered to a wide range of professionals*	*4*	
**Wider attitudes (what do you think?)**	**11**	**1**
*Positive or negative views of the training can impact implementation*	*7*	
*Compulsory implementation*	*3*	
Organisation taking ownership of making the required changes*	*1*	
**Spark further learning (light a fire)**	**7**	**0**
*Facilitate reflection*	*7*	
*Facilitate independent learning*	*0*	
**A strong alternative? (bigger and better)**	**7**	**0**
*Provides something that is missing or needed*	*4*	
*A better alternative to previous practice*	*3*	
**Post-training support (keep it going)**	**4**	**0**
*Follow-up progress checks with trainers*	*2*	
*Access to guidance resources*	*1*	
*Refresher training*	*1*	
Continued training	*0*	
**Implementation fidelity (by the book)**	**4**	**0**
*Regular utilisation*	*2*	
*Fidelity monitoring*	*2*	
**Resource availability (out of time)**	**3**	**0**
*Sufficient dedicated time*	*3*	
*Sufficient resources and staffing*	*0*	
**Surplus value (above and beyond)**	**2**	**0**
*Applicable to a wide variety of scenarios*	1	
*Surplus value*	1	

***Note*** : Starred (*) themes and codes are inductive. Those unstarred represent the categories and sub-categories from the review by Banwell et al. (2021) that were used to guide interview schedule production, and the deductive element of the present study’s qualitative content analysis

### The present study

SLRs are thorough collations of evidence often focused on a very narrow topic. It is therefore surprising that few researchers refer to these papers when designing their own studies [23]. Only 51% of respondents in a study by [24] stated that they consulted a meta-analysis when determining outcomes that warranted investigation in their research. Doing this can reduce ‘research waste’. Efficiency is crucial within the health field: one that often lacks research resources [24]. It follows that SLRs, as one of the most robust forms of research summary, “should be capable of directing all types of health research” [25]. These findings and observations indicate a clear need for more studies that evidence the value of synthesis papers [24].

Considering the above, it made logical sense that the findings of our review [22] could be used to guide this primary research in two ways. First, the directive points produced were used to build a schedule to interview a range of professionals who had undertaken GM i-THRIVE Academy training modules. Second, using a combination of deductive and inductive reasoning, transcript analysis was guided by the evidence-based synthesis factors, using them as indicative themes for what we could reasonably expect to see. This underutilised approach, whereby review evidence is tested against an active, current training intervention, had the potential to generate specific and relevant, yet evidence-based, recommendations.

The aims of the present study were to accomplish the following tasks:

- Establish whether the barriers and facilitators to training delivery and implementation reported in our review were present within the GM i-THRIVE Training Academy.
- Identify any additional barriers and facilitators present in the experiences of those completing GM i-THRIVE Training Academy modules, that were not evidenced in our review.
- As a result of the above two aims, generate tailored recommendations pertinent to GM i-THRIVE, to form part of a comprehensive evaluation of the programme’s implementation. The extent to which these recommendations can be applied to other training programmes will be reasoned and discussed.

## Methods

### Reporting guidelines

The production of this paper was guided by the Standards for Reporting Qualitative Research (SRQR) [26]. These guidelines consist of 21 criteria, developed to improve transparency in the reporting of qualitative studies. We have adhered to these by, for example, ensuring that the member checking process [27] was sufficiently described, researcher characteristics were explained, and that the data analysis process was comprehensively detailed.

### Design

The present study was a qualitative case study evaluation, involving semi-structured interviews with attendees of the GM i-THRIVE Training Academy modules. A directed approach to the qualitative content analysis was adopted. This meant that although our prior research findings [22] were used to guide analysis, there was also the potential for revealing additional knowledge [28]. This approach aligns well with a pragmatic epistemology. In essence, we focussed on the suitability and purpose of the employed research methods when choosing them, which did not necessitate thinking too abstractly about the construction of knowledge [29].

### Participants

Participants needed to have attended at least one of the four GM i-THRIVE Training Academy modules. Those eligible (N = 623) were approached by email. We attempted to vary the sample of participants by considering the following three factors:

1. The training module(s) that they completed.
2. Their professional role, namely whether they work within CAMHS, or within the wider workforce.
3. The Greater Manchester locality borough within which they work.

The factors were considered in that order of importance, in hopes of recruiting a suitably diverse sample, as is desirable for qualitative multi-site implementation research [30]. However, so that a suitable number of participants could be recruited, the above strategy was applied with flexibility, and a primarily opportunistic approach was adopted. Nine participants (Table 2) were eventually recruited. In terms of Greater Manchester locality, nine boroughs were represented, although two participants reported that their work took them across multiple boroughs. A third of participants (n = 3) represented one borough. Attendees of all four training modules were represented, with most participants having only attended one. Three participants attended face-to-face training sessions prior to COVID-19 lockdowns, whilst four attended virtual sessions once restrictions were in place. One participant attended sessions in both formats. Participant 6 had not attended a training module. Despite failing to meet the inclusion criteria, we decided that their data should be included owing to the valuable insights given regarding their inability to gain a training place. As the remainder of the interview schedule did not apply to them, only one extract from their transcript was included in the content analysis below.

**Table 2:** Characteristics of the participants recruited for the present study

Participant number	GM i-THRIVE Training Academy module(s) attended	Online or face-to-face training?	GM locality borough
**1**	All	Both	A
**2**	Building confidence in letting go and managing difficult endings	Online	Multiple
**3**	Getting risk support	Face-to-face	B
**4**	Shared decision-making; Getting risk support	Face-to-face	C
**5**	Building confidence in letting go and managing difficult endings	Online	D
**6**	Did not attend	N/A	Multiple
**7**	Getting advice and signposting	Online	E
**8**	Getting advice and signposting	Face-to-face	B
**9**	Getting advice and signposting	Online	B

***Note*** *: Professional roles were excluded from this table, and locality names were masked, to ensure anonymity*

### Researcher characteristics

The authors were externally commissioned by GMHSCP to conduct a comprehensive evaluation of i-THRIVE in Greater Manchester. Because the authors are affiliated with the University of Manchester rather than GMHSCP, the data analyses and conclusions drawn were unlikely to be biased by vested interest. The first author attended in-person training sessions to establish a feel for the content and format of the delivery. It is predicted that the structured nature of the data analysis method, and the authors’ impartial professional positions, contributes towards ameliorating the impact that any subjective opinions gained in these sessions might have. The data analysis process was primarily carried out by the first author, yet overseen and ‘sense-checked’ by the second and third authors. Whilst the latter authors did not receive the same immersive experience of GM i-THRIVE training of the first author, they were also free of any resultant biases. Their role was driven, therefore, by checking that analyses appeared logical, readable, and comprehensive: crucial characteristics of qualitative reports [31].

### Ethical considerations

The present study was categorised as a “service evaluation” by the NHS Health Research Authority (HRA). This was confirmed by both the HRA’s online decision-making tool [32], and the University of Manchester’s Research Ethics Committee (UREC)’s decision tool. This was in addition to verbal agreement from the commissioners of the evaluation of GM i-THRIVE. Consequently, the need for ethical approval was waived. The study was, however, informally reviewed and approved by the second and third authors (the first author’s supervisory team), and the study’s commissioners. Ethical principles such as obtaining full informed consent, ensuring anonymity, and stating a participant’s right to withdraw were followed. Participants were given a £20 voucher, to thank them for their time. All procedures within this research were performed in accordance with the British Psychological Society’s Code of Human Research Ethics [33].

### Data collection procedures

A semi-structured interview schedule was developed, guided by the nineteen categories from the aforementioned SLR findings [22]. Each category pertained to a barrier or facilitator of training delivery or implementation as identified through meta-synthesis (see Table 1). The 47 sub-categories, also presented in Table 1, further informed question generation, to ensure that these key factors were probed. The schedule consisted of 20 broad questions, overarching several prompts and sub-questions used to a varying degree depending on the detail and direction of the participants’ responses. All interview questions and prompts are included in the supplementary materials associated with this paper.

Owing to COVID-19, interviews were held using online conferencing software. Detailed study information sheets were provided, and consent obtained, prior to meeting. After transcription by the first author, each typed transcript was returned to the corresponding participants for ‘member checking’, to ensure that the transcript represented their interview, and to allow amendments or omission of any data that they no longer wish to be analysed [27]. Despite this task, we were careful to ensure that participants did not feel overburdened by the research process. For this reason, and in line with guidance by Elo et al. (2014) [31], who suggested that without full insight into the entire research process, participants cannot meaningfully validate final themes, we chose not to verify the post-analysis themes with our participants [31, 34, 35].

### Data analysis

Data were analysed using qualitative content analysis. Thematic analysis and content analysis are both suitable for studies with relatively descriptive research questions, that do not warrant deep and complex interpretation of meaning to answer [36]. However, the latter uses theme frequency as proxy for significance, concentrating more on surface features than assuming latent meaning [36]. This relatively objective, systematic method was considered more suitable given our study aims.

According to Hsieh & Shannon (2005) [28] the ‘directed’ qualitative content analysis approach is best suited to scenarios where prior knowledge of a topic exists, but the study aims to clarify or expand that knowledge. The findings of our recent SLR [22] form a strong evidential framework through which to explore i-THRIVE’s own training, thereby developing knowledge that could be truly meaningful and relevant to the programme. Using this directed approach, before the interviews commenced, a list of initial (deductive) themes were drawn, matching the nineteen concluding categories of our review [22] (see Table 1). The 47 sub-categories were treated as deductive codes. This was so that the transcripts could be checked for extracts corresponding to these. Since the findings of the review related closely to the interview schedule, this was deemed an appropriate way of cross-validating the review findings against our participants’ experiences. Any topics that appeared in the transcripts that could *not* be categorised with these initial codes were given a new code, allowing for a mixture of deductive and inductive code and theme generation. We could consequently ‘test’ the GM i-THRIVE interview data against the findings of the SLR [22], but still remain open-minded about the possibility of additional salient factors unaccounted for by the SLR.

When all interviews were complete, they were transcribed, member checked, then re-read to enhance familiarity. Using NVivo (version 12), the first transcript was read, and data were coded using the deductive codes and themes. Any ideas not suitably covered by a pre-existing code were added under a separate heading for inductive codes. Subsequent transcripts followed a similar process, although newly generated inductive codes were used alongside existing deductive codes when categorising extracts. Once all transcripts had been coded like this, they were read through once more, ensuring that all transcripts were considered with all inductive codes. All extracts relating to a certain code were considered together, to refine code titles, or to split extracts into further codes where necessary. Once coding was judged complete, codes referring to a similar barrier, facilitator, or other training element were grouped. They were then checked against the SLR’s list of categories (‘themes’) to establish whether they could be grouped under any of these. It was important not to force the data into the categories, so this was only done where it appeared suitable. These deductive themes were also modified or expanded as necessary, to encompass the content of any new codes added. As mentioned previously, the frequency of each code was noted, with frequent codes influencing, to a greater degree, how themes were worded, and the extent to which they were discussed. A subset of final themes, codes, and extracts were ‘sense-checked’ by the second and third authors. This process of verifying confirmability [37] by ensuring that data labelling and thought processes make sense, is a suitable way of adding rigour to qualitative research. Endeavouring to add validity, as we should with quantitative data, is neither worthwhile nor suitable.

## Results

The interviews were coded using 43 of the 47 deductive codes. However, all 19 deductive themes were represented within the data (see Table 1). 26 inductive codes emerged during this part of the analysis, of which 22 were grouped under the existing 19 deductive themes. Two new inductive themes were constructed with the four remaining codes, which were entitled “expectations versus reality” and “issues relating to the COVID-19 pandemic”. Table 1 shows the themes that represented the data, listed in order by frequency of extracts relating to each, and their accompanying codes. Inductive codes and themes are clearly marked in the table. Owing to the large number of themes, only a selection are discussed within this results section, guaranteeing that the analyses are sufficiently deep [38]. In line with principles of content analysis, whereby frequency indicates thematic significance [36], the three themes formed by the highest number of extracts were chosen for full analysis. These themes also, conveniently, contain the most inductive codes. Each one is therefore informed by a balance of deductive and inductive reasoning. Additionally, the two new inductive themes were chosen for full analysis, owing to their immediate relevance to GM i-THRIVE, specifically under the context of the COVID-19 pandemic. Participant numbers given after each supporting extract correspond to those in Table 2.

### Deductive theme 1: peer support (with a little help from my friends)

Trainees appreciated meeting and interacting with colleagues from diverse professional backgrounds. Opportunities to make professional connections were valued, through which a broad range of roles and experiences could be discussed. One way that this was facilitated was through group work. One participant, who attended in-person training, mentioned that the plethora of professional backgrounds and ways of working, that were made apparent when working through scenarios together as part of a group task, were beneficial to problem-solving.

*There was like a scenario, or a couple of different scenarios, that we looked at in the afternoon. Where people’s differences really came out in the way that we were all approaching the same challenge. You could really see different backgrounds and different kinds of professional training, and how that played out, and how we were all approaching it slightly differently. So it was really good to get many heads together. (Participant 3)*

Participants also mentioned specific elements of GM i-THRIVE, and how interacting with staff from other locations and professions allowed them to discuss experiences of implementing a certain concept. They could then take this knowledge back to their own workplace.

*There were a few examples from other localities about how they were using ‘Getting Advice and Signposting’ as a principle and how they were implementing it. It was good to have that thought process. It did apply to what we were trying to do. (Participant 7)*

It was also helpful to learn that issues and problems with implementing the changes were shared by others. This dialogue, of discussing these concerns with colleagues with the same professional goals, reduced feelings of personal failure.

*Because we were all in the job to help people out. So when we can’t, it’s quite difficult. But it was nice to know that nationally that happens. And that’s not a reflection on you as such. (Participant 5)*

Participants reported maintaining the links they forged during the training sessions. As a result, they gained a wider network of colleagues to contact and get support from.

*From that day, I’ve got better relationships and a better network of people that I personally would feel comfortable reaching out to. From that day*. *(Participant 3)*

### Deductive theme 2: does it reflect reality? (keep it real)

Participants desired more opportunities to discuss their own workplaces: to share unique perceived challenges and barriers with leaders and other trainees. A consultation-style system was recommended here. This would enable localities to present their own scenarios to leaders, who could then fill gaps in their thinking by suggesting specific ways to implement a concept.

*It would be good to have a smaller group or a breakout session, a bit like a consultation offer, as part of the training, where we could come up with our ideas. Then ask more specific questions and have that opportunity to have them ask us questions about things that we might not have thought about. That would have been useful. (Participant 7)*

In terms of whether trainees felt the training equipped them to deal with the diverse reality of their workplace, a mix of views were raised. Concerns were held about applicability to cases that presented the biggest professional challenges. Learning how to deal with complex, non-routine cases appeared to be a common training need, with one participant reporting that the training had limited applicability to the disengaged CYP that they worked with. As a result, they wished they had been given more information about how to utilise the GM i-THRIVE training in their work with these CYP.

*For me, the young people I work with are the most disengaged. So, it is quite difficult. The universal service doesn’t always fit, so things like 42nd Street, so brilliant, but for a lot of my young people, they won’t engage with it, they won’t go to it, they won’t go to appointments. And so, it’d be useful to just have more information about how to access support for those young people. (Participant 8)*

Training played a vital role in providing meaning to the whole implementation process of GM i-THRIVE. Ensuring that the programme remains visible and central was seen as vital in terms of sustainability.

*Keeping it live and meaningful, I think is really important. So I think those two connect. So in order to be able to kind of keep it sustained, you’ve got to be able to keep it live and meaningful in each locality*. *(Participant 9)*

However, whilst keeping GM i-THRIVE relevant and meaningful to trainees is crucial, deeper system change is also necessary. This is so that services, as a means of providing reformed care, are fully prepared to receive the programme. This participant felt that although the THRIVE model advocates a flexible mindset, the current structure of services, that are likely aligned to older models of provision, makes this new mindset difficult to apply.

*Mental health services aren’t as fluid as the model states they should be. That can be difficult to implicate sometimes. (Participant 5)*

### Deductive theme 3: suitability (know your audience)

Participants respected the difficulties of appropriately pitching training to such a diverse group of professionals. This is a pertinent issue for GM i-THRIVE, as an implementation with multi-agency working at the heart of its ethos. Despite these challenges, the training was reported as well structured, with concepts explained in order of complexity to aid understanding.

*It explained some basic theory about the approach. But in a way that you didn’t feel that it was too superficial or patronising. It then scaffolded a bit more and took you into more detail about the model. But I think you could just join it nicely at the level that it was. (Participant 3)*

Even those with an extensive level of previous work in the CYP mental health sector did not feel that the training was too simplistic. They felt that the knowledge obtained was timely and relevant.

*I think it was really well pitched for a really wide area. Although I’ve got, I don’t know, 15 plus years of qualified work, it didn’t feel like it was too basic, because actually, it was just building on, and adding kind of tools, which were really, really pertinent at the time, actually. (Participant 2)*

Some trainees, however, said that even though they were mental health trained, their position outside of the medical field made some of the language used in the training difficult to understand. It was consequently more difficult to imagine using the concepts in their work.

*A challenge from it has been some of the language used. I’ve not come from a medical background, and a lot of language feels very ‘medically’ and isn’t necessarily something that we understand. And you know, I find myself having to Google things, which is all my professional development, which is great. But I think that sort of can be a challenge. (Participant 4)*

Language was also mentioned in terms of how the training forged links between GM i-THRIVE and trainees’ own background knowledge, work, and other related training programmes. This emphasises the importance of the ‘common-language’ element of GM i-THRIVE, showing that understanding can be enhanced by unifying terminology. This is especially true where, as this participant states, similar concepts and theories are often explained differently by different training providers.

*Understanding the model helped me in the role that I was in at that point as well, to look at how it might link with other changes, in other languages. Because lots of different training was going on at the same time, and there were lots of changes in language. And I was really mindful that these things aren’t in competition. They’re all very much from the same kind of theoretical approach. But if I understand what the language means, in each of these different contexts, I’ll be able to make sense of it better. (Participant 3)*

### Inductive theme 1: expectation versus reality

In this inductive theme, participants expressed an assortment of motivations for attending the GM i-THRIVE training. These motivations moulded the expectations they had prior to attending, resulting in varying levels of satisfaction depending upon whether these expectations were met. Although the training was not mandatory, a small number of participants mentioned being asked to take part by senior colleagues. These participants tended to have fewer prior expectations of the knowledge or skills that they might gain, but this did not seem to influence their perception of its usefulness. In the extract below, the participant appreciated the insights into current ways of working and thinking within CYP mental health. They appeared optimistic about the changes that GM i-THRIVE hopes to make.

*I’m not even sure what I expected from it really, I guess because it wasn’t something I requested. It was just something that I was told to go on, but I enjoyed it because it was good to see what was going on in the background in mental health, and what plans that they were considering for young people over the next few years. Hopefully, there will be a lot of changes. (Participant 1)*

Most participants, however, had made a personal decision to book onto the training. Some described specific gaps in their own skills, or processes that they found difficult. They hoped that the training would help them to overcome these obstacles.

*I chose to attend it […] The thing I identified that I struggle with the most is discharging people and feeling sad about discharging people, or feeling bad, so it was good to get on it. (Participant 5)*

Another related motivation was to disseminate the learning to teams within a locality. This participant attended as a representative of their locality. They hoped to gain a deeper insight into the programme’s principles, that could then be translated back into their work.

*I thought it’d be useful to come along and see first-hand what the principles were and how it was articulated, then I could take it back into my role and articulate it in the same way […] the reason I came along to that one, again, through choice was to make sure that we capture all of the key principles of what that meant for young people and for families. And we could implement that in our hubs. (Participant 7)*

Many participants reported that the training exceeded their prior expectations. They readily mentioned the practical utility of the topics discussed, and as a result, how quickly they could transfer their learning to their work.

*I think it was definitely useful to come along. In terms of my expectations, they made things really clear about what the principles were and how they applied. So that sort of exceeded my expectations. (Participant 7)*

However, not all trainees felt that their expectations were met. Attending with a specific training need can lead to frustration and disappointment when this requirement is not actualised. This participant said that they had hoped to learn more ways to refer CYP, but instead felt that the training covered content that they already knew.

*I think I found it frustrating, really, because I think I wanted to have different pathways to refer young people. I felt like it was telling me how to refer. Whereas the problem is that the referral pathways are so limited. I know how to refer. And I know a lot of the organisations have just got massive waiting lists. So, I was hoping, I think, to get some extra pathways. (Participant 8)*

### Inductive theme 2: issues relating to the COVID-19 pandemic

Owing to social distancing guidelines enforced in the UK at the onset of the COVID-19 pandemic, in-person training modules were moved to an online format. Unsurprisingly, issues associated with this modality shift were frequently reported. The networking element of training was mentioned several times: better facilitation of group conversation would have been appreciated in online sessions, but participants acknowledged that the virtual training environment, by nature, made this difficult. Longer networking periods are less practical and useful when offered through video conferencing, and importantly, less pleasant.

*I think you were given around 10–15 minutes, which, when virtually, I really don’t think you can do much more can you, you lose like the networking side. (Participant 5)*

Participants were sympathetic to the fact that engaging trainees is harder online. Even though training was delivered well, the live virtual environment can never provide the same immersive networking experience as in-person meetings.

*With the ‘Getting Advice and Signposting’, it was delivered well over (Microsoft) Teams […] again, just having that opportunity to have conversations I think, was missing a bit. But that was just due to the nature of the way it was set up. (Participant 7)*

Completing training remotely often resulted in reduced focus, which was especially difficult for group work. It was very easy for people to turn off their cameras and disengage, with no consequence. Here, a resolution is suggested.

*You went into breakout rooms, and say there were five of you, sometimes it would only be three talking. Because two people would, you know, be off camera, and clearly not there! I don’t know how they could manage that differently really, apart from maybe putting facilitators in each breakout room, that could be a way forward for future, if it was going to continue to be done online. (Participant 1)*

When attending in-person training, trainees were united during breaks, meaning that focus on GM i-THRIVE topics was maintained for the entirety of the session. When attending virtually, it is easier to become distracted and distanced. Again, this is especially true during breaks, where trainees are likely to choose to complete other tasks rather than continue networking.

*On the online ones, it felt as if when there was a break, everyone scattered for half an hour and then came back […] So I didn’t really use that time to reflect on what I was doing that was related to the training […] Whereas if you’re in that space, where you’ve got all these other people in front of you and they’re all talking about THRIVE, even if you don’t have a conversation, you’ve still got that break to reflect on some of the learning and some of the practices that you do in your everyday work. (Participant 7)*

Finally, one participant mentioned problems with getting a training place, despite their keen interest. Whilst this was an isolated account, this highlights potential issues with the reach and access of the programme. The participant was also unaware that the programme continued online during the lockdowns.

*I was trying to book on […] And I just couldn’t. I just... didn’t get any details about it. So I filled in the form. And then I didn’t hear anything back, and then COVID happened. So obviously I never kind of chased it up after that. (Participant 6)*

## Discussion

In the present study, nine professionals from across Greater Manchester, UK, were interviewed to discuss their experiences with GM i-THRIVE training modules. This was to establish the typicality of the reported barriers and facilitators when compared to those identified within the existing literature [22]. In our earlier work, we synthesised nineteen practical categories based on previous literature (Table 1). By converting these nineteen directive action points into interview questions for the present study, evidence-led evaluations of the strengths and weaknesses of GM i-THRIVE’s training were undertaken, showing where improvements can be made, and which elements of the training have been delivered successfully. ‘Testing’ SLR evidence against an active, current training intervention for a piece of primary research is an underutilised method, yet one with potential for a robust, evidence-informed set of recommendations. We optimistically view this approach as the key strength of the present study.

As explained earlier, the 47 sub-categories of the SLR were treated as deductive codes, of which 43 were represented within the interviews. 26 new inductive codes were also produced, however only four of these could not be classified under the nineteen deductive themes of the SLR. The remaining 22 codes were thus incorporated into the deductive themes. This means that rather than reporting experiences entirely at odds with the literature, the participants’ reports were of a similar nature, and could therefore be used to *expand* the categories. When we look at specific examples of the inductive codes that were incorporated into the pre-existing themes (Table 1), they are not dissimilar in nature to the deductive. Rather, they appear to focus more narrowly upon one element of a deductive code. To provide an example of this, under the “peer support (with a little help from my friends)” theme, the deductive code “trainees from different professional backgrounds sharing ideas and experiences” shared the theme with inductive codes like “encourage conversation” and “learning about problems in the wider sector”. These two codes can clearly be conceptualised as the sharing of ideas and experiences, except that the specific experiences of the facilitation of discussion, and hearing about cross-sector difficulties, were raised, and therefore coded as such. This broadening of the thematic content resulted in those points receiving attention within the analysis.

As a conclusive statement on how closely the present study’s findings ‘match’ those of the SLR, we would assert that although very similar, the contextual nuances of the training programme meant that slight but important differences were seen. Given that every intervention, training or otherwise, has its own unique differences and circumstances, we would predict that using this evidence-driven interview design method in other studies, to examine other interventions, would lead to a similar outcome. Qualitative SLRs akin to ours [22] should therefore be treated as reliable yet broad evidence syntheses. The extent to which findings are treated as guidance should also reflect that. Implementers should, thus, not ignore the importance of speaking to those working with their own intervention, to consider the range of diverse experiences, contexts, and problems present within their teams. With more research effort given towards taking advantage of the deep and detailed investigative work of evidence syntheses, especially when designing primary research, it would be interesting to observe if this reasoning is true. The SLR and the present study, although interesting standalone pieces of research, can be treated as a ‘part one’ and a ‘part two’ of a combined investigation. The SLR served as a scoping mechanism through which to identify the questions that would yield the most valuable insights, with the present study going on to apply this knowledge.

There are several limitations to the methodology used in the present study that warrant discussion. A methodological paper [28] was used to guide the choice of qualitative content analysis used in the present study. Those authors acknowledged limitations to the directed method, which will now be addressed in turn. Although content analysis is a relatively systematic way of exploring qualitative data, a direct approach means that prior theory is used as a starting point in the process of sorting the data into themes. As much as we might consciously try to ignore the influence of our previous knowledge when using deductive codes, the confirmation bias caused by this knowledge is still, unavoidably, likely to influence our work. The data may then appear more likely to conform to these deductive codes. Although the processes of ‘sense-checking’, and of the development of inductive codes, may have ameliorated this bias somewhat, it is nevertheless worth considering the influence that biases, including the more general subjectivity bias that is so often raised as a weakness of qualitative research, may have had on this research. We appear, however, to be moving towards holding qualitative research to a different, yet just as rigorous, set of standards as quantitative studies [37]. Providing that it is acknowledged appropriately, bias should not necessarily be seen as a problem to overcome, rather it should be accepted as a core principle, and indeed a strength, of interpretative work [39]. Another limitation is that theory-driven analysis can lead to context being ignored [28]. We believe that our earlier discussion of the nuances associated with individual interventions, and the consequent deviation of participant accounts from pre-existing frameworks goes some way to addressing this. This is particularly true given that the present study, and the implementation of GM i-THRIVE, took place during the COVID-19 pandemic. We cannot expect previous research to match these unprecedented circumstances in any way.

In terms of limitations relating specifically to the present study, we note the relatively small sample size. Whilst the ideal sample size for qualitative research appears predominantly a matter of opinion [40], we nonetheless appreciate that a few more participants would have added strength to this study. Though, the eventual opportunistic nature of our recruitment did not allow that. Despite this, however, we believe that our sample was sufficiently homogenous for a robust picture of perceived barriers and facilitators, yet sufficiently diverse to capture a wide range of views and experiences (see Table 2). Examining divergent as well as convergent perceptions is a crucial element of multi-site implementation evaluations such as this [30]. In line with this, the closer focus on individual experiences allowed by a smaller sample can be viewed as a strength, and the value of these individual opinions and insights should not be downplayed.

The mixed deductive and inductive coding system resulted in a large number of themes, some of which were backed by only a small number of extracts. Initially, the fact that only a few of these could be analysed fully in this paper appeared concerning - perhaps the richness of the data would be lost if so many themes remained unexplored. Further reflection, however, led us to conclude that the direct and pragmatic nature of the interview resulted in extracts that often covered several concepts. Indeed, extracts were often coded more than once. Additionally, many themes are conceptually similar, and are often just different ways of focusing on a certain topic. These ideas were also highlighted in the SLR, where theming was guided by the framing of a concept as well as the content [22]. Ultimately, the way that the themes were built, in that several were conceptually similar, means that fully exploring more than a handful within this paper would have resulted in a great deal of repetition. As those themes chosen for presentation either contained the most extracts, or were completely inductive, it follows that they should form the backbone of the recommendations made, owing to their salience and relevance to GM i-THRIVE respectively.

Below, we present the recommendations for the GM i-THRIVE training implementors. Before that, however, it seems prudent to discuss the generalisability, or transferability, of the present study’s findings, especially in light of these recommendations. A key question is whether the findings can be applied to other training settings, particularly outside of the CYP mental health sphere. Without further in-depth investigation, we cannot state, either way, whether similar findings would emerge had the same interviews been given to staff receiving training in a different field. The perceived barriers and facilitators may, or may not, be universal characteristics that can be used to improve training across the board. However, given that transferability was not a central aim of this study [41], this should be done cautiously, with due consideration given to the context of our research. Nonetheless, we optimistically suggest that given the substantial and focussed nature of the SLR findings [22] that were used to guide this study, the recommendations that we make can and should be used to develop and improve other training programmes relating directly to the mental health of CYP. Indeed, qualitative meta-syntheses can be seen as a way of combining several investigations. This makes them easier to apply to practice and research, but also to enhance the transferability of the included studies [42]. Our focus on GM i-THRIVE as a case study frames our findings within a localised public health intervention. Thus, although the recommendations are worded accordingly, GM i-THRIVE can simply be seen as a good example of application to a relevant training intervention.

Based on qualitative investigation, we make the following recommendations for the continued dissemination of the GM i-THRIVE Training Academy. The citations within these recommendations relate to studies that were included in our SLR.

- Participants valued time to interact with others attending the training. Ensure that structured group dialogue can bring out the strengths and differences of each group member [43], and that they are aware of each other’s roles and where these roles fit into the wider system of CYP mental health provision in Greater Manchester.
- Unstructured peer interaction was also valued, and the advantages of interaction were lost in the online training environment [44]. Where training must continue online owing to the pandemic, efforts to ameliorate these issues, and facilitate discussion, should be made.
- Many participants wished for more opportunities to discuss the nuances of their own workplaces [45], and to reflect upon what elements of GM i-THRIVE would look like in their contexts. This is especially true for those working with CYP who are at the highest risk level. Providing as much applicable and tangible meaning to the training as possible will be valuable [44, 46].
- Participants valued the scaffolded structure of the training [47]. However, consideration should be given to fully explaining key words and concepts. Ensuring that language, terminology, and jargon are fully clarified at the start will maximise understanding by trainees of different professional backgrounds [48, 49]. By further reinforcing their ‘common language’ tenet, GM i-THRIVE can make the dissemination of their training more effective. Trainees will be able to make closer links between GM i-THRIVE and the practices and procedures that they already follow.
- Clarifying the aims of the training, and for whom it is the most suitable, will maximise satisfaction [50], especially when trainees are made aware of this in sufficient advance.
- It is vital that everyone who needs or wishes to take part in the training can do so [46, 51]. Some keen individuals may slip ‘under the radar’ owing to miscommunication or confusion about how to take part. Making the sign-up process easy to understand, and ensuring that managers are clear on how their staff can take part, will improve training reach.

## Conclusions

The present study examined semi-structured interview transcripts, the schedule for which were developed using a coding scheme devised from qualitative SLR findings [22]. Identified themes largely echoed those of the review, which provided a vital starting point in terms of the questions that needed to be asked, and the elements likely to be of interest in the data. Several important differences were also found, and it is plausible that these may reflect the contextual nuances of GM i-THRIVE itself, and of issues arising because of the COVID-19 pandemic. The study provides a valuable example of how qualitative evidence syntheses can aid study design and analysis. Studies following a similar research strategy will further demonstrate the utility of SLRs for guiding research: an approach that is, thus far, underused. Study limitations were discussed, and six key recommendations were made. We suggest that these findings are transferable to similar settings. Still, for optimal training effectiveness and efficiency, implementers should invest time and effort into considering the unique issues and challenges surrounding their intervention and trainee pool.

## Electronic supplementary material

Below is the link to the electronic supplementary material.

Supplementary Material 1: Interview schedule (see file Semi structured interview schedule.docx)

## Data Availability

The semi-structured interview schedule can be found within the supplementary materials. The dataset generated during the current study is not publicly available owing to privacy concerns owing to potentially identifiable information within the interview extracts. This is in accordance with the research governance policy of the University of Manchester. However, the data may be available from the corresponding author (EB) on reasonable request.

## List of abbreviations

A&E: “Accident and Emergency” (ER/emergency department)
CAMHS: Child and Adolescent Mental Health Services
CYP: children and young people
NHS: National Health Service
