# Supplementary material for: Barriers and facilitators to training delivery and subsequent implementation of a localised child and adolescent mental health initiative: a qualitative content analysis
Source: BMC Med Educ. 2023 Apr 19;23:264. doi: 10.1186/s12909-023-04238-9 (PMC10113980; doi:10.1186/s12909-023-04238-9)
Supplement: Supplementary file 1 — Supplementary Material 1: Interview schedule (see file Semi structured interview schedule.docx) [file 12909_2023_4238_MOESM1_ESM.docx]

Semi-structured interview schedule including prompts:

Hi, how are you? I’m Emily, a PhD student at the University of Manchester, and I’m working on an evaluation of i-THRIVE in Greater Manchester. Thank you for agreeing to speak to me today. As you’ll be aware, I’m going to run through an interview with you about your experiences with the THRIVE training academy. The questions won’t be too taxing, but we’re just keen to get your thoughts on it so that we can feed back to the team ways which it can be improved going forward as part of my evaluation of i-THRIVE. The first few questions will be about the training itself, what you thought about it, and the following ones will be about how, and to what extent, you have utilised the training in your job.

Try not to divulge any confidential information about anyone that you mention, if you do mention another person in whatever context. If you do mention any names though, they will be anonymised in the transcript. I just want to make it clear that even though I am working on an evaluation of i-THRIVE which is funded by them, my position is completely impartial, so please feel free to be as honest as you can with your experiences: we want to hear the good and the bad, and there won’t be any negative consequences of you doing this!

I just want to reinstate that you are free to stop and/or withdraw from the study at any time, if you just let me know, we can stop. If you do decide to continue, this Zoom call will be recorded. Zoom also automatically transcribes our conversation, and once I’ve used your recording to check this generated transcript for accuracy, this recording, both audio and visual, will be deleted. But, if you do want to turn the camera off now so that your face isn’t recorded at all, you can do that if you wish? It’s up to you. I’ll also set up the pseudonym feature on Zoom. Do you have any questions?

Are you happy to begin? I will start the recording if so.

***

**What is your job title?**

(If unclear from job title) what does your role involve, in a nutshell?

**Which Greater Manchester borough do you work in?**

**Would you describe the area you work in as urban, suburban, rural, etc.?**

**Which THRIVE academy training modules have you attended?**

1. Getting advice/signposting

2. Building Confidence in Letting Go and Managing Difficult Endings

3. Risk Support

4. Using i-THRIVE Grids to Improve Shared Decision Making

5. The THRIVE Framework: Leading system wide transformation

**When did you undertake the training approximately?** (If after first lockdown, clarify that they did training online)

**Overall, what were your motivations behind attending the training?**

- Did you choose to attend it, or was it mandated?
- What did you hope to gain?

***

**Now I would like to talk to you about the set-up of the training.**

**Can you describe the general set-up of the training? For example how many people were leading, and how was the event structured? Things like that!**

**Also…**

- Did you feel that the training was suitable and appropriate for you?
- How did your prior knowledge of CYP mental health prepare you for the training, i.e. did they assume too much prior knowledge? Was it too basic? Or perhaps just right?
- How relevant was the training to your job?
- How easy was it to imagine yourself using the training, when the leader was explaining it?

**Was the training accessible for you and your colleagues?**

- Perhaps think about access, location, use of technology…
- How did you feel about the length of the training session(s)…
- …and the amount of content that was covered?

**Can you tell me about the support you received, both during and after the training?**

**What qualities did the person/people leading the training have?**

- What did you feel they did that was helpful?
- Was there anything that you disliked about the approach taken by the training leader/leaders?
- Is there anything that they have done to be more helpful?
- If you can remember, what mechanisms were in place for you to ask questions, get feedback, etc. from the trainers?

**Thinking about the other people who attended the training…**

- Did you get a chance to meet them?
- How much time was given to meeting and working with other people?
- Was this useful?
- Why?
- (Depending on tone of answer) would you have preferred more of a chance to interact with them?
- Why?
- Have you kept in contact with any of the people who attended the training with you? How so/why/why not?

**Thank you! Thinking about the bigger picture in terms of your experience:**

- How did your hopes/expectations match up to the actual experience?
- Which parts exceeded them?
- What were you disappointed by?

***

**Thinking now about taking the training back to your work, how did people generally feel about the training in your workplace?**

- (If good or bad) what did people have to say about it?
- Did this impact your enthusiasm?

**To what extent has the training been useful to you?**

- Have you used any of the skills that you learned?
- Can you give some examples?
- What do you think would encourage you to use the training more regularly?
- If you have applied the training during your interactions with CYP, how did you find this?
- Was it easy? Why?
- What would have made it easier to apply the training?
- For example, was the training relevant enough to your role to use it properly? Was it flexible enough to use with the range of young people that you help?
- What challenges were there?
- If give vague/generic/too broad answers, give tailored example based on person’s job role and the training they did (e.g. can you give an example of how you gave advice to/signposted a young person based on what you learned? How have you made a joint decision with a young person about their ongoing care? Remember not to divulge anything about the young person that would make them identifiable).

**To what extent have the skills you learned in training replaced old ways of working?**

- Which skills/what have they replaced?
- Could you explain whether things have changed in terms of the way you do things as a result of the training? Is there a big difference/a small difference? If so, how do things differ…
- For example, has it changed the way that you signpost/give advice/make decisions alongside CYP/deal with the end of a CYP’s treatment (use appropriate example)
- **(If not much replaced),** why do you think this is? How did it compare to the old way that you did things?
- **If so,** why do you think this is? How did it compare to the old way that you did things?

**To what extent do the new knowledge/skills you gained fit in with your day to day role? For example, do you have time to use the skills you gained in an average interaction with a CYP?**

- What would make the new skills fit in better with your day to day activities? E.g. more time, a change in how the skills fit in with what you did already?

**Do your colleagues know about THRIVE?**

- Did many of them also go to the training?
- Do you think this has influenced the extent to which the training is used back at work? How? How do you think having your colleagues on board has affected you/your utilisation of the training?

How closely have you followed the specific guidance that you were given in the training? Why/why not?

Do you have any other observations or experiences about the training itself, or about its impact on your work, to share that you think we might find interesting?
